# On the Morbid Changes of Saliva

**Published:** 1853-07

**Authors:** A. Snowden Piggot


### ARTICLE IV.

*On the Morbid Changes of Saliva.* By Prof. A. SNOWDEN PIGGOT, M. D.

DR. SAMUEL WRIGHT, whose classification will be followed in this article, gives the following table of salivary diseases:

Deficient saliva.
Redundant saliva. (A spontaneous, β excited.)
Fatty saliva.
Sweet saliva.
Albuminous saliva. (A transparent, β white.)
Bilious saliva.
Bloody saliva.
Acid saliva.
Alkaline saliva. (A fixed alkali, β ammoniacal.)
Calcareous saliva.
Saline saliva.
Puriform saliva.
Fœtid saliva.
Acrid saliva. (A per se, β from foreign matters.)
Colored saliva.
Frothy saliva.
Urinary saliva.
Gelatinous saliva.
Milky saliva.

*Deficient Saliva.*—Saliva may become temporarily deficient from causes purely physiological. This secretion is subject to the same laws which govern all the others. Among these, none is better known than the arrest of the action of glands in consequence of some strong mental emotion. The insane, who live in a perpetual state of emotional excitement, are remarkable for the general torpor of the secernents. Narcotics and stimulants, which act especially upon the emotional centres, suspend the secretions.

A familiar example of this mode of action upon the salivary glands, is the extreme dryness of the mouth and throat, to which the public speaker who makes his maiden harangue, is notoriously subject. The rice ordeal among the Hindoos, is another illustration of the same fact. This is commonly employed for the detection of a thief, and is practiced in this way. The suspected parties are placed in a ring, the detector being in the centre. A handful of rice is then given to each individual. All are ordered to chew it for a given length of time and then to reject it upon a leaf or clean piece of bark. The different pellets are then examined, and should one of them be unmoistened by saliva, the unfortunate person from whose mouth it comes, is immediately seized as guilty of the crime alleged. This test may occasionally have perverted the ends of justice, particularly if the culprit were thoroughly convinced of the infallibility of the method.

Morbid deficiency of saliva may arise from obstruction of the ducts, from inactivity of the glands, and from disorder of the stomach. Obliteration of the ducts leads to the disease called ranula, which is a distention of a duct by its proper secretion on account of a closure of its natural outlet. This closure may be congenital or dependent upon tumors pressing upon the ducts or on calculi closing them. These will be considered in a separate article. We shall only say here that the sublingual gland is most subject to them.

A distinction must be made between common and encysted ranula. The contents of the former variety are only saliva as it comes from the glands; those of the latter are albumen, mucus and some salts, largely diluted with water. Gmelin states it to be water 94.6, solid matter, 5.4. The latter consist of albumen, extractive matter, and the salts of the blood.

Gorup Besanez has analysed the fluid of ranula with the following result:

| | |
|---|---|
| Water, . . . . . | 95.029 |
| Traces of fat and chloride of sodium, | 1.062 |
| Aqueous extractive matter, . . | 0.923 |
| Albuminate of soda, . . . | 2.986 |

Under the microscope, blood and exudation corpuscles, were observed, none of the ordinary characters of saliva appearing.*

Inactivity of the glands is common in old age, but is occasionally found in persons otherwise in full health and vigor. As it is a purely local affection, local stimulants will generally be sufficient entirely to relieve it.

Disease of the stomach, especially obstinate indigestion, is often accompanied by a disagreeable dryness of the mouth dependent upon salivary deficiency. It is usually relieved by tonics, assisted, if necessary, by local stimulants.

*Redundant Saliva.*—Redundant saliva may be *spontaneous*, the fluid remaining healthy; or *excited*, the secretion being depraved by an alteration in its constituents or by the presence of foreign matters.

It is present in infants sometimes from birth, sometimes even in the fœtus, but is most common as an accompaniment of dentition. It is not our purpose to go into a regular pathological history of these diseases. We consider them only as they bear upon the chemical history of this fluid, and as they throw light upon the various influences of the secretion on the teeth.

The specific gravity of the saliva in infantile ptyalism is 1003.1 to 1005. It is oftener deficient in ptyalin and in sulphocyanogen than the saliva of adults. Being generally produced in local asthenia, "it is liable to an occasional variation in quality, consisting in a predominance of its albuminous constituent;" I have known this, says Dr. Wright, "to equal 3 per cent."†

* Rees. Article, saliva—in Cyclopœdia of Anatomy and Physiology.

† Wright, *op. cit.*

Drivelling is a disease of the other end of life, and is eminently asthenic. It is also found in idiots. Wright says he has seen the specific gravity of the discharged saliva as low as 1001.1; often 1003, and never above 1005. "It is commonly clear and transparent like water, and seldom has the blue tinge which distinguishes the healthy secretion. It froths very little when agitated, is often deficient in albumen and contains less ptyalin and sulphocyanogen than natural; the latter I have more than once found to be wanting, though the usual saline constituents were present in their accustomed quantity. The secretion is sometimes alkaline, but oftener neutral."

Vogel has examined the saliva of spontaneous salivation and gives this table:

| | |
|---|---|
| Water, . . . . . . | 991.2 |
| Ptyalin, osmazone, fat and albumen, | 4.4 |
| Salts of soda, potash and lime, . . | 4.4—1000 |

Mitscherlich and Guibourt found no increase in the solid constituents, while the sulphocyanogen and ptyalin were deficient.

Though in gastric diseases the saliva is usually diminished, there are affections of the stomach which are accompanied by a great increase in the flow of saliva. Nausea, for example, is always attended by this symptom, so is hunger, so is appetite generally, especially when stimulated by the sight, the smell, or even the thought of a favorite dish. Pyrosis has been usually supposed to arise from a morbidly increased flow of fluid from the gastric glands, but Dr. Wright (*op. cit.*) gives reasons for believing that the flow comes chiefly from the salivary organs. He derives his arguments, first, from the specific gravity of the fluid which he quotes from Gilding Bird. The average specific gravity of the fluid of gastrorhœa and pyrosis was 1009.7, and that of saliva, 1008.1 The lowest saliva was 1004.3, the highest 1015.5, while the lowest pyrotic fluid was 1005.8 and the highest 1020.9. In the second place, he states, that the reaction in pyrosis is that of saliva. He experimented on nineteen specimens in which reaction was strongly alkaline in eleven, feebly alkaline in three, neutral in three, and acid in two. In every case the state of the saliva corresponded with that of the ejected fluid. This is strengthened, if we consider and appreciate the difficulty of supposing the stomach, which is engaged regularly in secreting an acid fluid, should suddenly change its action and make an alkaline secretion, and then again, without any apparent reason, return to acid secretion again.

One of Dr. Bird's objections to this theory is derived from the behavior of reagents. Sesquichloride of iron, according to this observer, did not give the characteristic blood-red tinge with pyrotic fluid but only an orange more or less deep. In reply to this Dr. Wright states that the sesquichloride rarely gives the blood-red color with saliva, unless an alcoholic solution of the dried secretion be used, because the animal matters present cloud the solution and obscure the tint. Ptyalin, he regards as characteristic of saliva, and he asserts that he has rarely made an examination of the liquid ejected in pyrosis, without finding a sufficiency of this principle to convince him of the native origin of the secretion. He gives the following analyses of Dr. Percy's as evidence in favor of his view.

No. 1.

| | |
|---|---|
| Water, . . . . . . . . | 938.4 |
| Solid matter consisting of mucus, chloride of sodium, free soda, and a trace of the matter upon which depends the peculiar, and characteristic odor of saliva, . . . . | 16.6 |

No. 2.

| | |
|---|---|
| Water, . . . . . . . . . | 994.1 |
| Ptyalin, . . . . . . . . | a trace. |
| Matter dissolved by alcohol, . . . . | 1.31 |
| Matter left after treating by ether and alcohol; mucus with a trace of albumen, | 3.63 |
| Saline matter; chlorides of potassium and sodium, with free alkali or carbonate, . | 2.88 |
| Loss, . . . . . . . . . | .08—1000 |

Dr. George Wilson has published an analysis of the fluid of pyrosis in which he coincides with Mr. Goodsir, in representing the most remarkable character of it to be the presence of a small cryptogamic plant, *sarcina ventriculi.* The organisms are in the form of square or slightly oblong transparent plates, of a pale yellow or brown color, and varying in size from the 800th to the 1000th of an inch. They were made up of cells, with dissepiments, which pressing against one another, became bulged out in the middle, so that the organism resembled a soft bundle bound with cord, crossing four times at right angles, and at equal distances. From these peculiarities, Mr. Goodsir gave it the name of *sarcina,* a wool-pack. The chemical reactions of the fluid differed from those just quoted from Dr. Wright. It was strongly acid and was found to contain free acetic, hydrochloric, lactic and carbonic acids.

*Ptyalism.*—Artificial ptyalism is most commonly produced by the preparations of mercury. The quantity of the liquid discharged, under the operation of this agent, is extremely variable. Its chemical composition has been variously stated.

Observers are especially divided as to the presence of mercury in this fluid. Bostock, Devergie and Wright could not detect it by the most delicate tests. Gmelin, however, obtained it by Smithson's method, which is a modification of the galvanic test. A large quantity of saliva was treated with nitric acid and evaporated. The dry matter was digested in nitric acid and dissolved in water, the fat was removed by filtration and a stream of sulphureted hydrogen passed through the solution. The precipitate obtained by this process contains sulphuret of mercury; it must be collected, digested in nitro-muriatic acid, evaporated, dissolved in dilute hydrochloric acid, and a bit of gold leaf enveloped in tin foil, or encircled by fine iron wire, suspended in the fluid. The gold is tarnished if mercury is present. No tin foil should be used which has not been itself tested for mercury. The tarnish must also be examined, for Taylor has shown that, in the presence of free hydrochloric acid, tin may, by galvanic action, be precipitated upon gold. The foil should, therefore, be made into a cornet and introduced into a reduction tube of narrow calibre. Heat being applied, if the stain be mercurial, a dew of minute metallic globules will collect upon the cool part of the tube.

Another mode of examination, more simple than this, is to let fall a drop of dilute nitric acid upon the stain. If it be mercurial, it is dissolved; if tin, it is rendered white and opaque from the formation of the oxyd of that metal.

Lehmann has always found this metal in the fluid of hydrargyria, both by dry distillation and by the galvanic test, his little battery being formed of a pair of very minute plates of copper and zinc, suspended in the acidulous solution. He believes the cause of failure in Wright and those who think with him, to be twofold: First, they have often examined the buccal secretion only, since, in the first stage of salivation, this constitutes almost the entire bulk of the sputa, the salivary glands being affected later; and secondly, unless the evaporation be conducted with very great caution, the mercury readily volatilizes with the aqueous vapor.

The specific gravity of the saliva, according to Wright, is increased prior to the occurrence of actual ptyalism. He attributes this augmentation of density to an excess of albumen, rarely of mucus, and regards it as the first indication of the action of mercury upon the salivary glands, preceding fetor and the tumefaction of the gums. The greatest specific gravity he met with in commencing ptyalism, was 1059. In one case in which violent salivation set in six hours after a dose of five grains of calomel, it was only 1004.8. After the discharge has fairly commenced, the density of the liquid diminishes and continues low till the action of the glands begins to diminish. Golding Bird estimates it at 1004.3, Thompson at 1003.8 and Wright has found it as low as 1001.5. When separated from the shreds of mucus which occasionally trouble it, it is nearly as limpid and thin as water. Generally as the excitement of the glands decreases, the secretion thickens and contains excess of fatty matter.

The sulphocyanide of potassium is very variable in its proportions; some specimens furnishing not a trace of it, while from others Wright obtained 3 per cent. In the majority of instances he observed an increase of sulphocyanogen.

The quantity of mucus is often unnaturally increased; frequently it is adventitious, being derived from the mucous membrane of the mouth, which becomes detached and is then partially dissolved in the secretions. "The specific gravity of the fluid is thus considerably heightened, and its transparency disturbed. The epithelium is at first suspended like nebulæ in the mass of fluid, but if left at rest, it gradually subsides as a greyish jelly-like sediment, and leaves the supernatant liquid comparatively clear. At other times the *proper* mucus of the saliva is unusually abundant, but the intermixture is so perfect, that, notwithstanding the increased amount of animal matter, there is no observable turbidity. The secretion is inordinately viscid, does not drop from the mouth of a bottle, but runs in a stream *(en masse)* and is not easily miscible with water. It does not froth more than common when agitated, nor does boiling furnish any extra coagulation. I have more than once, under these circumstances, remarked the entire absence of albumen."* Often, however, the saliva of mercurial ptyalism contains albumen in excess.

It is usually increased in alkalinity and thoroughly transparent, though sometimes, as already intimated, turbid from the admixture of mucus and epithelium. It froths greatly when agitated and does not recover its clearness readily by rest. It absorbs oxygen rapidly, decomposes easily, and rarely deposits any sediment till this last change has commenced.

The quantity of *ptyalin* is often increased, whence arises the sharp penetrating taste and smell for which this morbid saliva is so remarkable. Should pus mingle itself with the liquid, *ptyalin* disappears and the pungent odor is lost. When *ptyalin* is abundant, the saliva decomposes with unusual rapidity, evolving an excess of ammonia. The reaction is almost constantly alkaline, and Wright makes it an element in prognosis, the change of this fluid from acidity to alkalinity, in febrile and inflammatory affections, being often the first sign of amendment. The exceptions to alkalinity are scrofulous and scorbutic cases, and excessive salivation. In the latter instances, the secretion becomes exceedingly offensive, and loses its characteristic properties. It is usually of a dark brown or fawn color, some-

* Wright, *op. cit.*

times intermixed with blood. When first discharged, it is opaque from clouds of mucus and epithelium, which are gradually deposited as a bulky sediment, and the supernatant liquor is left clear and of a brownish or reddish color. "The secretion seldom contains *ptyalin* and is often also deficient in sulphocyanogen. It readily decomposes and then becomes additionally offensive and very ammoniacal. Its odor appears to depend upon a fatty matter which is separable by ether, and by distillation. This saliva bears little resemblance to the natural secretion, except in being furnished by its glands. It exerts no digestive action upon starch, and is very liable to produce nausea or vomiting, if swallowed."*

L'Heritier gives as the mean of three analyses of the fluid of mercurial ptyalin,

| | | | |
|---|---|---|---|
| Water, . . . . | 970.0 | in place of | 986.5 |
| Organic matters, . . | 28.6 | " | 12.6 |
| Inorganic matters, . | 1.1 | " | 1.9 |

The mean amount of ptyalin was 2.6. The increased quantity of organic matter he accounts for by the increased action of the buccal mucous membrane.

Simon† has published an analysis of saliva from a patient who had just concluded a mercurial course of four weeks duration. It had an acid reaction, occasioned by the presence of free acetic acid. It was very viscid, of a yellow color and possessed a sickly, disagreeable, acid smell. No mercury was found in it. After evaporation to dryness, all the acid reaction had disappeared, showing absence of lactic acid. It contained a very large quantity of semifluid fat, a considerable amount of albumen and traces of caseous matter. Many fat vesicles, epithelium cells and so called salivary corpuscles were visible under the microscope. 1000 parts contained:

| | |
|---|---|
| Water, . . . . . . . . | 974.12 |
| Yellow viscid fat, . . . . . . | 6.94 |
| Ptyalin, with extractive and traces of casein, | 3.60 |
| Alcohol extract, with salts, . . . . | 7.57 |
| Albumen, . . . . . . . . | 7.77 |

* Wright, *op. cit.* † Animal Chemistry, 302.

"The salts consisted of a large preponderance of the chlorides of sodium and potassium, associated with the lactates of soda and potash and with a small quantity of the earthy phosphates. On contrasting this saliva with the normal fluid, we are struck with its large amount of solid constituents, arising not from any increase of the ptyalin, but of the fat, the extractive matters, the albumen and the salts."

Dr. Wright gives three analyses of this fluid:

| | No. 1. | No. 2. | No. 3. |
|---|---|---|---|
| Water, | 989.8 | 988.7 | 987.4 |
| *Ptyalin*, | 1.7 | 1.9 | 2.7 |
| Fatty acid, | a trace. | .4 | .7 |
| Albumen, with soda and albuminate of soda, | 1.5 | .6 | 1.3 |
| Mucus, with a trace of *ptyalin*, | 2.1 | 3.8 | 3.5 |
| Lactates, Muriates, Phosphates, } Potash, Soda, Lime, | 1.9 | 2.4 | 2.6 |
| Hydrosulphocyanates, | 3. | 2.2 | 1.8 |
| Loss, | 1000. | 1000. | 1000. |

In the analysis, No. 1, the sulphocyanide of potassium was separately estimated and found to be .3 in the thousand parts.

Iodine stands next to mercury as a general sialogogue. The mercurial fœtor has been stated by some observers to be present in this form of salivation, while others deny that there is either fœtor, sponginess of the gums or loosening of the teeth. The explanation of this discrepancy will probably be found in the facts recently elicited by M. Melsen's researches. This accurate and careful observer shows that mercury may remain a very long time in the system, in consequence, probably, of its property of forming insoluble compounds with the various organic substances of which the body is composed; and that iodide of potassium is capable of dislodging it and making it soluble, so that it will again enter the current of the blood, and may, consequently, if set free in sufficient quantity, produce its primary effects of salivation, erythema, &c. Dr. Budd relates a case in which, five months after taking mercury, a patient was violently mercurialized in this way, by iodide of potassium.

The tendency of iodine and its compounds to pass off through the salivary secretion, has already been mentioned. Like most other substances, however, it may change the organ of elimination and may be discharged through the skin, the kidneys, or even by means of the fluid evacuated in consequence of the operation of a seton. Under circumstances like these, its elimination by the salivary glands is of course checked, if not entirely suspended.

Pure iodic salivation differs materially from that produced by mercury. The vascularity of the mucous membrane of the mouth and of the salivary glands may be unchanged, but oftener it is increased, the glands especially becoming tumid and tender. The secretion itself contains an unusual amount of mucus or albumen, the excess of one or other of these elements constituting its chief deviation from the healthy state. The unpleasant taste of iodine is often perceived in the secretion, and weight at the root of the tongue, aching of the jaws and singing in the ears are complained of. An increased flow of tears and of nasal mucus generally accompanies this form of salivation.

Other halogens, especially chlorine and bromine have been known to salivate. Ptyalism is one of the effects of arsenic even when given in medicinal doses. Lead and antimony are said to act in the same manner. The terchloride of gold is an undoubted sialogogue, and some obstetricians even fancy that rubbing the gums with metallic gold facilitates dentition and increases the quantity of the salivary secretion. A host of other substances have been asserted to exert the same action. Among them are found, opium, conium, belladonna, nux vomica, digitalis, xanthoxylum, hydrocyanic and nitric acids. Sulphur increases the quantity of sulphocyanogen, as already stated, and sometimes after the protracted use of this agent, sulphureted hydrogen has been liberated by the addition of an acid to the saliva. The action of local sialogogues in increasing the alkalinity of the saliva has been already noticed.

*Fatty Saliva.*—In addition to the fatty matter found in healthy saliva, adventitious fat is often present in this secretion, which is consequently changed in appearance and properties.

The quantity of fatty saliva secreted is usually less than that of the healthy liquid; it is freer and more abundant in the evening; it has a greasy taste and feel in the mouth; when depraved, it is often very offensive and is sometimes compared to castor oil; it imparts a sticky or slimy sensation to the whole mouth, and is with difficulty rejected. In three cases, examined by Wright, from whom this description is copied, the specific gravity was 1009.8, 1010.7 and 1011.3. It is usually frothy on the surface, and the bubbles disappear very slowly. Its color is a dull or yellowish white of variable intensity, never semitransparent and bluish like healthy saliva. Its smell is greasy and sickly, never normal. The *ptyalin* is sometimes altogether wanting and the sulphocyanogen. It absorbs oxygen sparingly and exerts but a feeble action upon starch. It imperfectly converts it into gum and produces no suga.

Wright's method of analysing this variety of morbid saliva was carefully to dry the specimen and exhaust the residue with sulphuric ether. On evaporating the ethereal solution we have fat, *ptyalin* and sulphocyanide of potassium. The two last are dissolved by washing in cold water and pure fatty matter remains, which must be collected, dried and weighed.

His analysis of what he considered an excellent specimen of fatty saliva is,

| | | |
|---|---|---|
| Water, | | 987.4 |
| *Ptyalin,* | | .7 |
| Adventitious fatty matter and fatty acid, | | 3.9 |
| Albumen, with soda and albuminate of soda, | | 1.5 |
| Sulphocyanide of potassium, | | a trace. |
| Mucus, | | 2.4 |
| Lactates, Muriates, Phosphates, | Potash, Soda, Lime, | 1.8 |
| Loss, | | 2.3 |
| | | 1000.0 |

This state of the saliva accompanies disorders of the alimentary canal, whether these are primary or consequent upon some other affection. In the saliva of a phthisical patient, Landerer found a great number of small fat globules aggregated into a viscid mass. These globules exhibited the properties of oleic acid. Wright found it present in phthisis, chlorosis, diabetes, jaundice, small pox, the dyspepsia of gluttony and of intoxication, and in poisoning by ergot of rye. Should it be persistent, he regards it as pathognomonic of disordered function of the lining membrane of the stomach and bowels.

*Sweet Saliva.*—This is a disease which is noticed in most of the larger systematic works on the practice of medicine. It is found in conjunction with a variety of morbid affections. The same state of the system which gives rise to diabetes may produce it, and indeed the two affections are often coincident. But it often occurs independently of the urinary disorder and in subjects, in other respects, perfectly healthy. Robust, active children and adults are sometimes attacked with it, in the early part of the day, particularly if the stomach be empty. It more frequently, however, is found in conjunction with gastro-intestinal irritation and other forms of depraved digestion and assimilation.

"It is either of a light fawn or a dead white color, with mucous nebulæ; froths easily by agitation, but its bubbles are not permanent; scarcely affords a coagulum by heat; is either acid or neutral to test paper; has a mucous or syrupy smell, which is increased by an elevation of temperature, decomposes readily and furnishes acetic acid."

"It imparts a sense of sweetness to the tongue; not always agreeable, but, for the most part, mawkish or sickly; sometimes followed by a secondary taste of bitterness, like woody nightshade; and again, by a sense of astringency. It is very adherent to the tongue and mouth, and its impression is often lasting. On its accession it is merely complained of, but its continuance is often very annoying and occasionally it nauseates distressingly."

It is analysed, by first filtering as usual, and after separating the *ptyalin* and fatty acid with ether, as before directed, passing distilled water through the filter to complete exhaustion. The saccharine matter is only partially soluble in water, and part of it is therefore found in the filtrate with the chlorides. The rest must be obtained from the residue by exhaustion with boiling alcohol. The chlorides are decomposed by acetate of silver, the liquid filtered from the chloride of silver, the filtrate carefully evaporated to dryness, and the dry residue weighed. The sugar and acetic acid are then got rid of by incineration, the pure soda left must be weighed, the acetate of soda allowed for, and the sugar estimated by difference. On fermentation with yeast, the aqueous solution yields a notable quantity of alcohol and on evaporation is reduced to a syrup.

Wright's analysis of sweet saliva is,

| | | |
|---|---|---|
| Water, | | 986.9 |
| *Ptyalin,* | | .3 |
| Fatty acid, | | .2 |
| Muco-saccharine matter, | | 5.6 |
| Albumen, with soda and albuminate of soda, | | .4 |
| Mucus, with a trace of *ptyalin,* | | 2.6 |
| Sulphocyanide, | | a trace. |
| Lactates, Muriates, Phosphates, | Potash, Soda, Lime, | 1.9 |
| Loss, | | 2.1 |
| | | 1000.0 |

The fluid analysed above was obtained from a diabetic patient in whom a spontaneous ptyalism had temporarily occurred.

*Albuminous Saliva.*—The normal variation in the quantity of albumen in saliva is set down by Wright at 0.2 per cent. for the minimum, and .5 for the maximum. Any proportion of albumen beyond these limits either way, constitutes disease.

Albuminous saliva may be classed under two heads, the transparent and opaque varieties. The first of these is almost perfectly transparent, colorless, and untroubled by nebulæ. It has less *ptyalin* and more sulphocyanogen than the healthy secretion, is very tenacious, froths exceedingly when agitated, and coagulates at 212°. Its specific gravity and alkalinity are great. It decomposes easily, becoming first turbid, then mouldy and ammoniacal. Its action on starch is not so decided as that of healthy saliva. It produces the same quantity of gum but less sugar and lactic acid.

The opaque variety has a high specific gravity, varying from 1016.8 to 1009.5. It is milky in appearance, absolutely opaque, and, when boiled, coagulates in flakes which subside, leaving a supernatant fluid like whey. There is but a small quantity of ptyalin and sulphocyanide of potassium in this variety of albuminous saliva. It is always strongly alkaline, has a mucous or mouldy smell, froths with permanent bubbles when agitated, absorbs but little oxygen and little action on starch. During decomposition, it evolves hydrosulphuret of ammonium, and sometimes hydrocyanic acid. The quantity of albumen varies. In four specimens, Wright found .61, .96, 1.01 and 1.03 per cent., respectively. The salivary glands are always in a disordered or sluggish condition when this albuminous saliva is excreted, and often digestion is more or less seriously impaired. Mercury and iodine, especially the last, produce this disease of the secretion. The drunkard and the glutton are peculiarly liable to it.

*Bilious Saliva.*—The bile, when once absorbed, or not eliminated by the liver, tinges, as we know, all the fluids and many of the solids of the body. The saliva does not escape the general contamination.

"Bilious saliva chiefly occurs in two forms, colored and colorless; more rarely it is met with containing only cholesterine.

"Colored bilious saliva is of various shades, from a golden yellow to a deep olive. The lighter specimens are generally alkaline, the darker are not uncommonly acid. The specific gravity of this saliva is greater than natural; its smell is sickly and offensive; its taste bitter and nauseous; it froths easily when agitated, and coagulates abundantly by boiling; protracted ebullition renders it ammoniacal and deepens it hue; it contains only a minute trace of *ptyalin* which usually has the color of the original fluid; sulphocyanogen is generally wanting; it exerts scarcely any action upon starch; it readily becomes putrescent, and then evolves either ammonia or its hydrosulphuret."*

Wright has analyzed it and gives the following as the contents of a single specimen, the only one, apparently, which he examined:

| | | |
|---|---|---|
| Water, | | 986.7 |
| *Ptyalin*, | | .5 |
| Fatty matter and fatty acid, | | 1.3 |
| Biliary matter, | | 3.2 |
| Cholesterine, | | .4 |
| Albumen with soda and albuminate of soda, | | 1.9 |
| Mucus, | | 1.6 |
| Carbonates, Muriates, Phosphates, | Potassa, Soda, Lime, | 2.3 |
| Loss, | | 2.1 |
| | | 1000. |

His process of analysis was, first to remove the ptyalin and fat by ether, then to exhaust the residue with boiling alcohol. By carefully evaporating and crystallizing, he then separated the cholesterine; he then extracted the biliary matter, leaving the chloride, by digesting absolute alcohol upon the residue of the last process.

"Colorless bilious saliva, as its designation is intended to signify, is free from any appearance of intermixture with biliary matter. Still it is never so transparent as the natural secretion and has either a dead white or a slightly dingy aspect. It is sometimes bitter, but more frequently imparts a mouldy taste to the tongue. It is always alkaline, with an abundance both of albumen and mucus. Its sulphocyanogen is deficient though rarely wanting; *ptyalin* is present in rather less proportion than natural, and its odor is not recognizable in the saliva, whether cold or hot. The addition of nitric or muriatic acid produces, after a few minutes or a few hours, a dull yellow

* Wright *op. cit.*

color, which gradually deepens to a faint olive. Protracted boiling, and spontaneous decomposition, give rise to the same effect in an inferior degree.

"This salivation will convert a small quantity of starch into gum, but it never generates any sugar."

"Saliva which contains cholesterine, free from intermixture with biliary matter, is of rare occurrence. I have seen it only twice, in one instance accompanying dyspepsia with hepatic derangement, and in the other succeeding to an attack of jaundice. In the former case, it lasted for three or four days; in the latter for about a day and a half. The quantity secreted was scarcely more than ordinary, but my attention was directed to it from the patient's complaining of a greasy taste in the mouth.

"This saliva is white and shining, and more dense than ordinary; it has an alkaline reaction, does not redden a persalt of iron, and is nearly odorless. Its albumen is in excess, but its saline constituents are in small proportion; it possesses feeble digestive properties, and is slow of decomposition."

*Bloody Saliva.*—This is a rare form of disease and one of no great consequence to the chemist, however important it may be to the practitioner of medicine. Its color depends entirely upon the condition of the hematin. It varies from a brilliant red to a deep brown or black. The first variety is darkened by the various gases which similarly affect arterial blood, while the second is not perceptibly brightened by oxygen. Its specific gravity is greater than that of the healthy secretions, its taste bitter, nauseous, saline or insipid. It is deficient in *ptyalin* but usually contains the normal quantity of sulphocyanogen. It is darkened by decay and during the decomposition evolves ammonia. It absorbs oxygen sparingly and is possessed of but feeble digestive powers.

*Acid Saliva.*—The acids with which the saliva is at present known to be contaminated, are the acetic, lactic, hydrochloric, oxalic and uric. It is a matter of great practical importance to ascertain the presence of acid in the saliva, as it exerts so powerful an action over the teeth, corroding them with extreme rapidity.

Acid saliva may have a sour or an exaggerated salivary odor, both of which are increased by heat. It reddens litmus-paper with greater or less intensity. It has about the same specific gravity as the healthy fluid, and sometimes presents an opaque appearance from the coagulation of its albumen. Its *ptyalin* is in the natural proportion, its mucus and sulphocyanogen commonly in excess.

In analysis, the acetic and hydrochloric acids may be separated from the salts and most of the organic matters by careful distillation and then neutralized by an alkali, evaporated and estimated from the salt. Lactic acid is estimated in the ordinary process of analysis. Some of it may be left on the filter. This is removed by ether with ptyalin and fatty acid. Water separates it with the ptyalin from the fatty acid, and the acid is then precipitated from the concentrated aqueous solution by acetate of zinc. Uric acid is left on the filter.

Dr. Wright makes a distinction between the *secretion* and the *excretion* of acid by the salivary glands, attributing the former to some disturbance of the glands themselves or of the digestive apparatus, the latter to some general disorder of the entire system.

"It rarely happens," says he, "that the salivary glands, when in a state of healthy activity, perform an excernent function, to free the blood from any temporary impurity, unless the organs proper for this task shall fail in discharging it. And then, even, if the material, oppressive or poisonous to the blood, be capable of being neutralized by any of the constituents of the saliva, the salivary glands are rather disposed to furnish these constituents in increased quantity to correct the offending matter, than to suffer a deterioration or suspension of their characteristic function in becoming partially or entirely emunctories. This rule, however, only applies to the salivary apparatus in a healthy condition; when diseased or disordered, spontaneously or by sympathy, the reverse generally happens."

In corroboration of this opinion, Dr. Wright cites several experiments, which, indeed, appear to be conclusive. He found on injecting various acids into the veins of healthy dogs, that an immediate and great increase took place in the quantity of the secreted saliva, and that its *alkalinity* was very much augmented. The saliva became acid in one case just before death. In one instance only, out of more than twenty experiments, did the salivary glands excrete the injected acid. The subject of this observation was a strong bull-terrier dog, into the veins of which, had been thrown three drachms of pyroligneous acid, diluted with six ounces of water. At first the saliva became very frothy and alkaline, continued so for six minutes, at the expiration of which time it was discharged very copiously and found to contain acetic acid. The pytalism continued for four hours and it was not until seven hours had elapsed that the saliva had resumed its normal alkalinity.

On the other hand, in diseased or feeble animals, the injected acids were usually discharged through the salivary glands.

Acidity of the saliva may depend upon a spontaneous derangement of the secreting glands. This is usually preceded by the ordinary symptoms of increased vascularity, pain, fulness, tingling, &c. It may be intermittent or continued. Stimulating gargarisms have commonly the effect of restoring the secretion to its normal character of alkalinity.

It may also depend upon a general condition of acidity in the system. This accompanies scrofula and other cachexies and depends upon a variety of circumstances which we cannot at present consider.

Disease of the stomach and bowels is another cause of acid saliva. Donné, who was the first to call attention to this pathological fact, says he never knew a single instance of this derangement in the salivary secretion, in which the functions of the stomach were healthily performed. This state of the saliva may be absent in some functional disorders of the alimentary canal, but is always present in inflammation of that important tract.

"I have never seen," says Dr. Wright, "a case of gastroenteritis, whether in a severe or a mild form, in which the saliva was not acid; and I have remarked that one of the first and most favorable indications of recovery is the return of the saliva to a neutral or alkaline state—I have known a patient to suffer for days from an attack of mild gastro-enteritis, and his saliva to be strongly acid, without his consciousness of it, and yet a sudden aggravation of his gastric symptoms to render the saliva almost immediately intolerable from its extreme acidity. On the other hand, I have heard a patient who complained not less of the excruciation and smarting in his mouth than of the burning heat and pain in his stomach, declare the acidity and acid taste of his saliva to be gone in a few minutes after the application of leeches to his epigastrium. The same effect is frequently produced by the counter-irritation of sinapisms and blisters. Nay, I have known the saliva of a gastro-enteritic patient to have a strongly acid reaction before the application of a blister to the epigastrium, and to be equally strong in its alkalinity during the time such blister was in operation."

"The saliva is impregnated with *lactic acid* chiefly in gout, rheumatism, ague, diabetes and gastro-enteritis; with *acetic acid* in aphtha, scrofula, scorbutus, small-pox, protracted indigestion and after the use of acescent wines; with *muriatic acid* in simple gastric derangement from immoderate or improper animal food, and with *uric acid* in gouty affections. When oxalic acid exists in the saliva, its presence will most likely be dependent upon depraved digestion or imperfect assimilation.

Acidity of the saliva is apt to occur in various other general and local disorders, particularly in fevers, both of the typhoid and inflammatory types; in measles prior to the eruption, and often subsequently; in miliary fevers, when the acidity is sometimes so excessive as to corrode the gums and impart a sensible roughness to the teeth; in maniacal cases, during the exacerbations of which the acid impregnation is often remarkably increased; in phthisis, in protracted venereal disease, in many skin diseases, in amenorrhœa, ricketts, catarrh, mumps, quinsy, cancer affecting any part of the digestive apparatus, worms and in the tedious dentition of weakly or scrofulous children."

*Alkaline Saliva.*—This variety dependent on excess of soda differs little in external character from healthy saliva. It has a mucus smell, and does not give the red tint with iron until after neutralization with an acid. It contains excess of albumen, and when the alkali is greatly superabundant a deficiency of *ptyalin* and sulphocyanogen. It acts less powerfully on starch than the healthy fluid, decomposes readily and becomes mouldy and ammoniacal.

It is possible that this condition of the saliva may accompany an excess of carbonate of soda administered medicinally. It certainly attends the injections of this salt into the veins. It is remarkable, however, that soda, the alkali peculiar to saliva, is alone eliminated through the salivary glands, the other alkalies and alkaline earths never being found in it, but always being discharged through the common emunctories. Experiments with mixed carbonate of soda and carbonate of potash were very instructive. The saliva and urine both became alkaline, but the former contained the soda, the latter the potash.

Neuralgia and nervous disturbance generally have a remarkable connexion with alkalinity of the saliva. In neuralgia, nervous tooth-ache, and ear-ache, this alkalinity is observable, especially on the affected side. Nervous disorders of the stomach, liver and other remote organs are also subject to the same law. Epilepsy is also accompanied by alkaline saliva, in some instances, so decided as to impart an unpleasant taste to this liquid. In hysteria, the discharge sometimes amounts to ptyalism; it is of a low specific gravity, limpid and feebly alkaline. In the excitement or exacerbation of mania, the saliva is frequently in a state of morbid alkalinity. The secretion itself may be either lavish or limited. I once saw the fury of a madman almost instantly subdued by the sudden accession of a profuse ptyalism, which continued for nearly three hours. The fluid was very strongly alkaline. In many cases of mania, the saliva is remarkable for its acidity. Its continued secretion excoriates the lips and gums and has been known even to corrode the teeth. In such instances there can be little doubt that the function of the stomach is considerably deranged."*

*Wright, *op. cit.*

Ammoniacal saliva is rare. It is dingy and clouded, with a very strong alkaline reaction. The odor is ammoniacal or disagreeably mucous. Fixed alkali, sulphocyanogen and *ptyalin* are all wanting. It possesses no digestive property. The alkalinity is dissipated by heat, on account of the volatilization of the ammonia. It is secreted in less quantity than usual and is excessively offensive to the taste. It is difficult to swallow and clings to the mucous membrane of the mouth.

It indicates a cachectic state of the system. Wright saw it in putrid fever, in scurvy, and in purpura hemorrhagica.

*Calcareous Saliva.*—The normal proportion of phosphate of lime is about 6 parts in every 1000 of saliva. This proportion may be morbidly increased, and then carbonate of lime is also present. Saliva containing this abundance of calcareous matter deposits it either on the teeth, or in the excretory ducts of the different salivary glands.

This variety of saliva is usually opaque, slightly frothy and white, like milk. Through the microscope it looks curdy. When the proportion of lime is considerable, the fluid, after standing, deposits a copious precipitate, leaving the supernatant liquid clear. It may be acid, alkaline or neutral.

In a case of mollities ossium, Dr. Wright found 1.4 per cent. of phosphate of lime in the saliva.

*Saline Saliva.*—The quantity of salts contained in saliva is small, but constant, two of them only being subject to variation. These are sulphocyanide of potassium, which is liable to diminution, and chloride of sodium which may be increased.

Excess of this salt in the saliva may depend upon an increase of the natural amount present in the blood. Injection of chloride of sodium into the veins of an animal always produces this condition of the salivary secretion. Persons who eat much salt are subject to an increase of this constituent in the saliva. If much liquid is taken, the salt is eliminated through the skin, and in some persons, whose cutaneous transpiration is very active, it passes off with great rapidity by this outlet. Men working in salt mines and manufactories are often covered on the surface of their bodies and especially their foreheads, with crystals of this salt which has transuded. Dr. Wright says, that he once examined the men in a large salt warehouse, and found that those whose skin was impermeable to the saline particles, suffered from a constant salt taste in their mouth, and their saliva was impregnated with chloride of sodium, while those, whose skin allowed a ready transit to the salt, were rarely troubled with saline saliva or with thirst.

It may also be produced by a purely local and idiopathic disorder of the salivary glands. Functional disturbance of the digestive apparatus, too, produces it.

*Puriform Saliva.*—This is nothing but saliva with an admixture of pus, and is, of course, to be recognized as purulent discharges always are. These have been already spoken of, and therefore need no further allusion to them in this place. We shall only say that it has a greater specific gravity than normal saliva, and more albumen; that it is always alkaline; seldom deficient in the characteristic elements of the natural secretion, that it is easy of decomposition and feeble in its digestive action.

*Fetid Saliva.*—Various strongly scented substances taken into the system, either at the mouth or by cutaneous absorption, may be absorbed and communicate their peculiar odor to the saliva. The term, fetid saliva, however, is not applicable to these conditions of the secretion, but to a morbid alteration of its constitution which may depend upon either local or general disturbance.

This variety of saliva according to Wright, is always turbid and flocculent, variable in color but usually of a red, brown, green or yellow hue; exhaling a putrid or cheesy odor, greasy and sticky to the touch; deficient in ptyalin and sulphocyanogen; either acid or alkaline; having little affinity for oxygen and scarcely any digestive properties. Its color and smell are due to fatty matter which is separated by ether or boiling alcohol. Tiedemann and Gmelin suggest that some phosphorus they found combined with this fat may communicate the unpleasant odor to it. Wright, however, was unable to find uncombined phosphorus in the saliva. It can hardly be necessary to add that this fluid not only does not assist, but, by its nauseous properties, absolutely impedes the function of digestion.

*Acrid Saliva.*—No fact is better known than that serious morbid changes may take place without any discoverable alteration, anatomical or chemical, in the solids or fluids of the body. So too, saliva may be gravely disturbed without any discoverable chemical change. The saliva of maniacs is often so acrid as to excoriate those parts of the body with which it comes in contact, and yet analysis reveals the natural constituents in their due proportion. Dr. Wright thinks this saliva capable of communicating hydrophobia. One thing is certain, bites of animals and even of men inflicted in fits of furious passion, have produced this terrible malady. If we consider also that the most careful examination of hydrophobic saliva has failed to reveal any alteration in the chemical composition of this fluid, we shall have but little difficulty in arriving at the same conclusion with Dr. Wright, that the saliva, thus unnaturally active, differs from the healthy fluid only in possessing in an extraordinary degree those properties which are peculiar to it.

*Of Colored Saliva*, it is not necessary to speak at any length. Indigo and some other coloring matters are capable of tinging this fluid. Acetate of lead imparts to it a distinct bluish tint. In the advanced stages of fever and in purpura, the saliva is often darkly blue. Dr. Wright suspects this to be due to Prussian blue formed in consequence of the decomposition of the blood.

*Frothy Saliva* does not differ from the healthy fluid in chemical composition. Its only peculiarities are its appearance and its undue viscidity. It is pathognomonic of nervous excitement, being found in hydrophobia, epilepsy, &c.

*Urinary Saliva.*—Dr. Prout records a case in which, the urine being greatly diminished, ptyalism came on. The saliva had a urinous taste and an alkaline reaction; was opalescent, slightly ropy and foamed when agitated. Its specific gravity was 1005.5. The soluble salts of lead, mercury and silver, and the mineral acids produced precipitates in it. Dilute acetic acid caused a precipitate, but none could be obtained afterwards by the addition of ferrocyanide of potassium, showing the absence of albumen. The analysis of Dr. Prout yielded the following results:

| | |
|---|---|
| Water, . . . . . . . | 991.35 |
| Animal matter peculiar to saliva, . . | 3.33 |
| Alcoholic extract, apparently similar to that obtained from the blood, . . . | 1.06 |
| Sulphuric acid, . . . . . . . . | .90 |
| Hydrochloric acid, . . . . . . . | .75 |
| Phosphoric acid, . . . . . . . . | .06 |
| Alkali, partly potash and partly soda, . | 2.55 |
| | 1000.00 |

"The urine of this woman was of an amber color, and slightly opaque. Its specific gravity was 1013.1. It contained crystals of uric acid and reddened litmus paper more strongly than usual. It contained much less urea than natural, but a large proportion of a brown animal substance, which appeared to render it very prone to decomposition, especially when exposed to heat. With a view of increasing the flow of urine, diuretics were given. These produced the desired effect. The urine was indeed more copious and natural, while the salivary discharge was proportionally diminished." The saliva here was evidently vicarious.

Dr. Wright records a case of granular degeneration of the kidneys, in which, on the suspension of the urinary secretion, vicarious ptyalism, set in to an amount varying from a pint and a half to two pints and a quarter in the twenty-four hours. "The secretion was of a fawn color, viscid, and loaded with films and nebulæ which finally settled into a heavy, opaque deposit. Its odor, at first putrid, became ammoniacal in a few hours, and the patient complained that its taste was salty and urinous. It was not reddened by a persalt of iron, and it furnished not a trace of *ptyalin.* An alcoholic extract of its dried residue was moderately diluted and treated with nitric acid, when crystals of nitrate of urea were immediately deposited. The proportion of urea never exceeded .5 per cent., but until the kidneys resumed their function, neither the salivation was diminished nor was the saliva free from the presence of urea. Directly, however, that the urinary secretion was re-established, the action of the salivary glands and their product became perfectly healthy.

Dr. Wright also records a case of ascites, arising from subacute peritonitis, in which urea was detected in the saliva. The saliva was of a pale chocolate color, slightly ammoniacal in odor, alkaline in reaction and disagreeable in taste. The quantity discharged in twenty-four hours was fourteen ounces and a half. No urine was secreted for three days after the ptyalism occurred. From a pint and a half of this saliva ten grains of urea were obtained by the usual methods.

*Gelatinous Saliva.*—Gelatinous saliva somewhat resembles gum water in appearance; it is imperfectly transparent and somewhat dingy, viscid and tremulous when cold, but becoming more fluid and clear on the application of heat. It does not easily froth when agitated, decomposes easily and becomes mouldy and sour. Its taste is mawkish and its smell greasy, its sulphocyanogen and *ptyalin* diminished in quantity, its specific gravity varying from 1000.9 to 1010.1. Its reaction is neutral or faintly acid; it absorbs oxygen sparingly and possesses little or no digestive power. It occurs in a debilitated and depraved state of the system.

Dr. Wright's analysis reveals the following as its constitution:

| | |
|---|---|
| Water, | 987.2 |
| *Ptyalin,* | .6 |
| Fatty acid, | .8 |
| Gelatin, | 3.6 |
| Albumen, with soda and albumate of soda, | 1.3 |
| Sulphocyanide, | a trace. |
| Mucus, | 2.5 |
| Lactates, Muriates, Phosphates, } Potash, Soda, Lime, | 1.7 |
| Loss, | 2.3 |
| | 1000. |

*Milky Saliva.*—This form of saliva is a metastasis from the mammary glands, and may take place either at the commencement of arrest of the secretion, or at its close as a critical flux.

It is an opaque white fluid, sometimes uniform in appearance but often curdy. Acetic acid increases the coagula. The secretion is healthy except with the addition of the constituents of milk. Its specific gravity is high, reaching, according to Wright, 1012.5. It is faintly alkaline or neutral and is easily decomposed. It readily absorbs oxygen but possesses very feeble digestive properties.

*Changes of the Saliva in Disease.*—We have but little to add to what has already been said upon this subject. We have already noticed the acidity of this fluid in certain inflammatory affections and functional disturbances of the alimentary canal. Another peculiarity of the saliva in inflammatory diseases is a diminution of the normal quantity of water, as may be seen from the following mean of six analyses made on the saliva in cases of inflammatory fever, pneumonia and erysipelas. For facility of comparison the table of healthy saliva constructed by L'Heritier an average of ten analyses is placed beside this result.

| | In inflammation. | In health. |
|---|---|---|
| Water, . . | 968.9 | 986.5 |
| Organic matters, | 30.0 | 12.6 |
| Inorganic matters, | 1.1 | .9 |

The proportion of ptyalin was found to be increased.

In *chlorosis*, saliva suffers from watery degeneration, in the same manner as the animal tissues and secretions generally. In *dropsy* with albuminous urine, the saliva was found by L'Heritier to contain,

| | |
|---|---|
| Water, . . . . . | 985.9 |
| Organic matter, . . . | 13.6 |
| Inorganic matter, . . | .5 |

Scherer has published an analysis of the saliva of a girl of fifteen years of age, who labored under a *scorbutic* disease of the mouth. There was copious ptyalism, the discharge from the mouth amounting to forty ounces in twenty-four hours. The secretions were very liquid, fetid and alkaline. Its specific gravity was 1004.

The following is the result of the examination of this fluid:

| | | |
|---|---|---|
| Water, . . . . . . . | | 988.8 |
| Solid constituents: | | |
| A caseous-like substance, precipitable by acetic acid, . . . . | 6.5 | |
| Fat taken up by ether, . . . | 0.6 | |
| Extractive matter and *ptyalin*, | 1.8 | |
| Carbonate of soda, . . . . | 1.2 | |
| Chloride of sodium, . . . | 0.7 | |
| Phosphate of lime, . . . . | 0.4 | 11.2 |
| | | 1000.0 |

On examination with the microscope, immediately after its discharge, the fluid was found to contain a large number of infusoria and confervoid growths.

